# Using remote sensing to quantify the additional climate benefits of California forest carbon offset projects

**DOI:** 10.1111/gcb.16380

**Published:** 2022-09-12

**Authors:** Shane R. Coffield, Cassandra D. Vo, Jonathan A. Wang, Grayson Badgley, Michael L. Goulden, Danny Cullenward, William R. L. Anderegg, James T. Randerson

**Affiliations:** ^1^ Department of Earth System Science University of California, Irvine Irvine California USA; ^2^ Black Rock Forest Cornwall New York USA; ^3^ CarbonPlan San Francisco California USA; ^4^ Department of Ecology and Evolutionary Biology University of California, Irvine Irvine California USA; ^5^ Institute for Carbon Removal Law and Policy American University Washington District of Columbia USA; ^6^ School of Biological Sciences University of Utah Salt Lake City Utah USA

**Keywords:** additionality, carbon offsets, improved forest management, nature‐based climate solutions, remote sensing

## Abstract

Nature‐based climate solutions are a vital component of many climate mitigation strategies, including California's, which aims to achieve carbon neutrality by 2045. Most carbon offsets in California's cap‐and‐trade program come from improved forest management (IFM) projects. Since 2012, various landowners have set up IFM projects following the California Air Resources Board's IFM protocol. As many of these projects approach their 10th year, we now have the opportunity to assess their effectiveness, identify best practices, and suggest improvements toward future protocol revisions. In this study, we used remote sensing‐based datasets to evaluate the carbon trends and harvest histories of 37 IFM projects in California. Despite some current limitations and biases, these datasets can be used to quantify carbon accumulation and harvest rates in offset project lands relative to nearby similar “control” lands before and after the projects began. Five lines of evidence suggest that the carbon accumulated in offset projects to date has generally not been additional to what might have otherwise occurred: (1) most forests in northwestern California have been accumulating carbon since at least the mid‐1980s and continue to accumulate carbon, whether enrolled in offset projects or not; (2) harvest rates were high in large timber company project lands before IFM initiation, suggesting they are earning carbon credits for forests in recovery; (3) projects are often located on lands with higher densities of low‐timber‐value species; (4) carbon accumulation rates have not yet increased on lands that enroll as offset projects, relative to their pre‐enrollment levels; and (5) harvest rates have not decreased on most project lands since offset project initiation. These patterns suggest that the current protocol should be improved to robustly measure and reward additionality. In general, our framework of geospatial analyses offers an important and independent means to evaluate the effectiveness of the carbon offsets program, especially as these data products continue improving and as offsets receive attention as a climate mitigation strategy.

## Graphical abstract

Improved forest management (IFM) is a common form of carbon offsetting, where landowners receive credits for maintaining carbon stocks above a business‐as‐usual baseline. We used remote sensing datasets to investigate “additionality” in the IFM projects within California. We developed a framework for comparing project areas to similar control forests to estimate whether the carbon being stored in the projects is additional to what would otherwise occur.

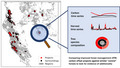

## 1 INTRODUCTION

Nature‐based climate solutions (NCS) include land management, reforestation, and conservation activities to sequester carbon, and are a component of most pathways to keep the planet below 1.5–2°C of warming (Roe et al., 2019; Smith et al., 2016). Compared to other carbon dioxide removal technologies, NCS are comparatively low cost (Psarras et al., 2017), immediately ready for large‐scale deployment (Minx et al., 2018), not reliant on energy inputs (Smith et al., 2016), and frequently come with environmental and social co‐benefits (Seddon et al., 2020). NCS have received increasing attention in the United States and internationally, for example through the U.S. Department of Energy's Carbon Negative Earthshot initiative to remove carbon, discussed at the 2021 COP26 summit (Gardner, 2021).

Among NCS, improved forest management (IFM) is estimated to have the greatest potential to reduce atmospheric carbon, through a combination of sequestration and avoided emissions. Surveys report IFM sequestration potential of up to 16 Gt CO_2_/year of negative emissions globally by 2030 (Griscom et al., 2017) or about half of total NCS sequestration (Fargione et al., 2018). Forest management practices that improve carbon storage include extending time between harvests, thinning to increase productivity, or increasing the stocking of trees. However, recent research has also highlighted the need for improved estimation and verification of the carbon potential of IFM (Kaarakka et al., 2021), which may be overestimated (Reise et al., 2022).

NCS, and IFM in particular, are a prominent component of California's climate mitigation policies. Administered by the California Air Resources Board (CARB), the California cap‐and‐trade program sets a cumulative carbon emissions limit, with tradable annual budgets that shrink each year, for large entities responsible for about 75% of the State's emissions. Emitters can use verified carbon offsets to comply with program requirements, subject to a set volumetric limit between 4% and 8% of their covered emissions (Haya et al., 2020). Offset projects occur across the continental United States and Alaska, in six categories: forestry, urban forestry, dairy digesters, destruction of ozone‐depleting substances, mine methane capture, and rice cultivation. While forestry offsets represent only 29% of all CARB offset projects, they account for 85% of all carbon credits issued so far (California Air Resources Board, 2021). Of the forestry projects, most (91%) are IFM projects, which are the focus of this study. According to state law, a central principle of these carbon offsets is that they must be “real, permanent, quantifiable, verifiable, and enforceable” as well as “in addition to” any climate benefits that would otherwise occur (California Health and Safety Code § 38562(d), 2011).

Determining whether carbon sequestration is *additional* is a central challenge for offset programs such as California's. Additionality is defined broadly in the Intergovernmental Panel on Climate Change (IPCC) Fifth Assessment Report as “beyond a business‐as‐usual level, or baseline” which is “difficult to establish in practice due to the counterfactual nature of the baseline” (Allwood et al., 2014). Baselines can be defined by a variety of approaches, usually involving estimation of average carbon stocking and expected economic constraints for the given land type and species assemblage; projects are thought to be providing *additional* benefits if they accrue carbon beyond the baselines' average rate. Some studies have criticized the counterfactual and hypothetical nature of baselines, which are impossible to prove (Murray et al., 2007). California has defined the term additionality in a way that is similar to the IPCC definition, referring to activities that “result in GHG removal enhancements [that] are not required by law, regulation, or any legally binding mandate applicable in the offset project's jurisdiction, and would not otherwise occur in a conservative business‐as‐usual scenario.” A “conservative” scenario is one that is “more likely than not to understate net [climate benefits]” (California Code of Regulations 17 § 95973, 2012; California Code of Regulations 17 § 95802, 2012); in other words, a conservative baseline should err toward higher baseline carbon stocks in forests to avoid over‐crediting.

**FIGURE 1:**
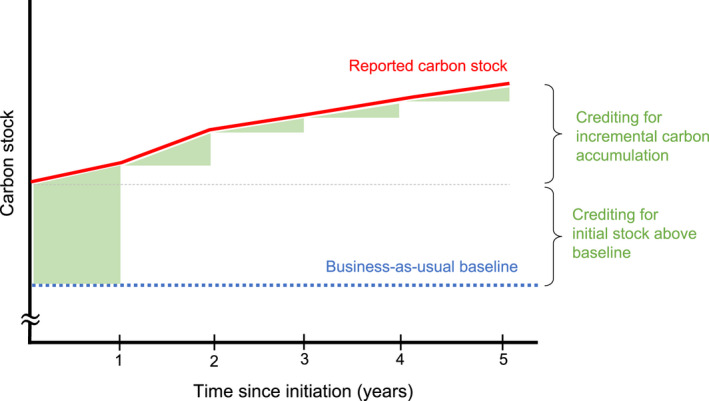
Conceptual diagram of carbon crediting following project initiation. California Air Resources Board (CARB) issues credits to offset projects after each reporting period. For the majority of improved forest management (IFM) projects, the first set of credits issued is mostly for initial carbon stocking above the baseline in the first reporting period, with incremental credits awarded thereafter based on carbon accumulation (minus estimated secondary effects and leakage which we do not discuss here). In this example, assuming negligible secondary effects and leakage, the project would receive a similar number of credits attributable to initial stocking and incremental accumulation after 5 years.

California Air Resources Board's offset program operationalizes these requirements through its Compliance Offset Protocol for U.S. Forest Projects (California Air Resources Board, 2015). According to the Protocol, additionality is quantified relative to a 100‐year static business‐as‐usual baseline calculated based on either (1) regional‐ and species‐aggregated U.S. Forest Service (USFS) Forest Inventory and Analysis (FIA) stocking levels or (2) a project's on‐site carbon stocking, depending on the condition of the project timberlands when the project enters the program. Projects are then issued carbon credits for any sustained carbon stocking above the baseline, usually a combination of the initial stocking above the baseline plus incremental growth in subsequent years (Figure 1). This approach for carbon crediting assumes that any carbon accumulation above the counterfactual baseline scenario would not have occurred without the offsets program; in other words, the protocol treats the baseline as true and assumes that landowners would otherwise reduce carbon stocks to baseline levels.

CARB's system of quantifying additionality has received scrutiny around how baselines are determined and whether carbon stocking above a baseline represents carbon accumulation that would not have otherwise occurred. Because for most projects the baseline depends on regional average carbon stocks, crediting is therefore sensitive to how those regions (“supersections” and “assessment areas”) are defined. Badgley et al. (2021) point out that strategic placement of projects on lands whose species composition is not well represented by the assessment area average has led to an average over‐crediting of nearly 30% (Badgley et al., 2021). Recent reports from investigative journalists suggest that some projects are non‐additional in their entirety, for example because they preserve forests that are not in danger of logging (Elgin, 2020; Song & Temple, 2021). Quantifying additionality is necessarily an imperfect process, based on unobservable counterfactual scenarios, and these examples support the idea that closer scrutiny and analyses, beyond what exists in CARB's protocol, could be implemented to help ensure true climate benefits (Anderson‐Teixeira & Belair, 2021).

In this study, we present a robust framework for systematically assessing additionality based on remote sensing ecosystem observations, and use it to investigate the climate benefits of the 37 IFM compliance offset projects within California. By comparing carbon and disturbance trends in offset project lands to those of nearby forest areas over the same period, we can infer whether the carbon being sequestered in project lands is additional to what may have been sequestered without the offsets program. This analysis also allows us to investigate CARB's IFM protocol assumption that, absent offset payments, carbon stocks would follow the baseline scenario. We present hypotheses regarding the signal expected from the presence of additionality in Table 1, considering information about pre‐project stand conditions (Hypotheses 1–3) and post‐project changes (Hypotheses 4 and 5). The first and fourth hypotheses are aimed at capturing evidence of carbon‐positive management practices; that is, management to directly increase the rate of carbon accumulation from what it would otherwise be. The second, third, and fifth hypotheses are aimed at capturing evidence of management to prevent degradation; that is, by extending rotation lengths or protecting existing carbon stocks in stands that would otherwise be at risk of harvesting.

**TABLE 1:** IFM forest offset additionality hypotheses.

Quantity	If carbon is additional	If carbon is not additional	Exceptions/caveats
1. Pre‐project *carbon* accumulation	Long‐term historical carbon accumulation rate has been near‐zero or negative; flat baseline is a realistic and conservative “business‐as‐usual”	Historical carbon accumulation rate has been positive; flat baseline likely underestimates the current “business‐as‐usual”	Historical carbon accumulation rate has been positive but is no longer expected to remain positive for most forests
2. Pre‐project *harvest*	Project areas were harvested at similar rates as other similar forests over recent decades	Harvest rates were high in project areas relative to similar lands before projects began, and forests are now recovering	Project lands are particularly productive and naturally have high harvest and high growth rates
3. Pre‐project *species composition*	Project areas have similar tree species to nearby forests, or have more high‐value species, indicating average or high risk of timber harvest	Project areas have less valuable species than nearby forests, making them less valuable and less likely to be at risk of timber harvest	Project lands have less valuable species but would otherwise be replanted with high‐value species; which species are considered “high‐value” may change over time
4. Post‐project change in *carbon* accumulation	Carbon accumulation rate after project initiation is greater than the pre‐project rate and greater than the rate for similar forests	Carbon accumulation rate after project initiation is similar or less than the pre‐project rate and the rate for similar forests	Carbon accumulation slows in the short term due to management like thinning to reduce fire risk
5. Post‐project change in *harvest*	Harvesting rate has decreased relative to pre‐project levels and relative to similar forests	Harvesting has stayed the same or increased relative to pre‐project levels for similar forests	Some carbon that will remain stored in wood products over 100 years is still additional

*Note*: These hypotheses describe the general characteristics of a portfolio of IFM projects that generate carbon additionality in an idealized offset program, considering rates of carbon accumulation, disturbance, and species composition.

Abbreviation: IFM, improved forest management.

Through our analysis, we also demonstrate the potential utility of remote sensing‐based geospatial data products as components of large‐scale carbon accounting and offset verification, especially as these products continue improving. Remote sensing products have been increasingly used for climate mitigation applications in the United States (e.g., Tang et al., 2021) and for tracking Reducing Emissions from Deforestation and Forest Degradation (REDD+) in the tropics (Bullock et al., 2020; Sangermano et al., 2012; Tang et al., 2019; West et al., 2020). The forest carbon and disturbance datasets we use here offer spatially extensive coverage, frequent temporal sampling, increased measurement transparency, and the potential for near‐real‐time monitoring of changes on the ground. Therefore, remote sensing could enable reliable, independent tracking and carbon accounting in offset projects, lower costs and barriers to entry for smaller landowners, and provide greater confidence and accountability toward large‐scale deployment of carbon offsets in and beyond California.

## 2 METHODS

### 2.1 Datasets

#### 2.1.1 IFM offset projects

We compiled documentation for all 37 active or previously active IFM compliance offset projects in California from two CARB‐approved registries: the American Carbon Registry (ACR, https://acr2.apx.com/myModule/rpt/myrpt.asp?r=111, accessed September 1, 2021) and the Climate Action Reserve (CAR, https://thereserve2.apx.com/myModule/rpt/myrpt.asp?r=111, accessed September 1, 2021). Access to offset project documentation is also available through the Air Resources Board Offset Credits (ARBOCs) issuance map (https://webmaps.arb.ca.gov/ARBOCIssuanceMap/, accessed September 1, 2021). From these registries, we obtained project landowner information, geographic polygons of project boundaries, the number of credits issued, and total carbon stocks for years where estimates are provided. We used the ACR and CAR project IDs to label projects in this study.

The carbon stocks provided in project documentation are self‐reported by offset project landowners who often work with carbon developers, consulting foresters, and third‐party carbon verifiers on implementation and reporting. Carbon stocks for each reporting period come from a combination of forest inventory and an approved set of empirical‐based forest growth models, as described in Appendices A and B of the Compliance Offset Protocol for U.S. Forest Projects (California Air Resources Board, 2015). Inventory methodology varies across projects, with CARB providing minimum requirements for field measurements which must take place every 12 years while providing detailed documentation of methodology. The aboveground live (AGL) carbon is estimated using CARB‐provided allometric equations based on diameter and height measurements. Between inventories, projects may apply approved models such as the USFS Forest Vegetation Simulator to grow tree diameter and height from the most recent inventory data. These growth models generally apply empirically derived rates of succession for different tree species following disturbance and management but do not incorporate climate impacts such as the ongoing drought on growth projections (e.g., https://www.fs.fed.us/fvs/). Given that current offset projects are less than 12 years of age, we expect most documented carbon changes to be based on these growth models.

**FIGURE 2:**
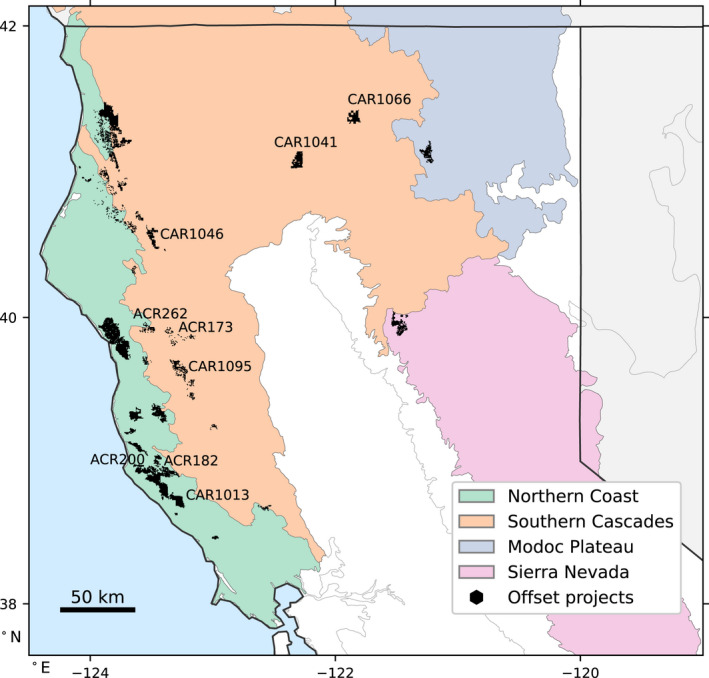
Study area encompassing improved forest management (IFM) compliance projects in California. We tracked 37 projects spanning four supersections as defined by California Air Resources Board (CARB) using U.S. Forest Service ecosections: Northern Coast (green), Southern Cascades (orange), Modoc Plateau (blue), and Sierra Nevada (pink). Nine of the largest and longest‐running projects are labeled.

We converted carbon stocks from units of ton CO_2_ to ton C by scaling by the molar ratio of 12.01/44.01. To compare against remote sensing data, our primary variable of interest from the registries was the AGL carbon component, but AGL was not consistently available. For the 12 projects that did provide AGL carbon stocks, the AGL carbon stocks were on average a factor of 0.806 ± 0.002 of the total carbon stocks (Table S1). Therefore, we scaled total carbon stocks by 0.806 to estimate the AGL component in the other 25 projects. Projects begin as early as 2012, with credit issuance beginning in 2013. The 37 project boundaries span four different “supersections,” defined by CARB based on ecosections or combinations of ecosections from the USFS (McNab et al., 2007; Figure 2).

#### 2.1.2 Remote sensing‐based carbon and harvest

We obtained and compared data for AGL forest carbon from two related geospatial data products which leverage remote sensing data and were available annually at 30 m × 30 m for 1986–2017 in California. The first dataset, from the Environmental Monitoring, Analysis and Process Recognition (eMapR) lab (Kennedy et al., 2018), is described as an “observation‐based, empirical carbon monitoring system” derived from a mix of field measurements, airborne lidar data, Landsat time‐series imagery, and statistical modeling. According to eMapR documentation, a time‐series algorithm (LandTrendr, Kennedy et al., 2010) was used first to detect changes in annually aggregated Landsat imagery and build maps of disturbance and stabilized surface reflectance imagery. The time‐series increments of stabilized surface reflectance imagery were then matched with FIA plot data using a gradient nearest neighbor (GNN) algorithm based on similar spectral, climate, topographical, and disturbance history characteristics to create yearly maps of FIA‐based forest metrics. Metrics including canopy height were then converted to aboveground biomass using allometric equations. The system was prototyped in the conifer‐dominated forests of the Western Cascades region of western Oregon and a small portion of northern California, but the final data are available across the contiguous US. The authors note little bias in tracking biomass densities, until high densities beyond 450–500 Mg/ha (210–235 ton C/ha) where biomass begins to be underestimated. They also show that the eMapR biomass estimates generally agreed well with inventory plot data at broad scales, with some noise at the 30 m × 30 m pixel level. For our study, we aggregated the data to compute project‐level means, with a mean project size of approximately 46 km^2^ (or 51,000 individual 30 m pixels).

The second dataset, from the Landscape Ecology Modeling Mapping and Analysis (LEMMA) lab (Bell et al., 2018; Ohmann & Gregory, 2002), is based on a similar approach as eMapR, using LandTrendr and a GNN model to match 30 m × 30 m pixels to similar inventory plots based on environmental variables (climate, geology, topography) and three Landsat Tasseled Cap indices. LEMMA varies slightly from eMapR in the spectral and environmental indices used, and the area over which the dataset was developed—in the case of the LEMMA California biomass product, over all of California and western Oregon.

The raw data for both eMapR and LEMMA have units of aboveground forest biomass per hectare, which we converted to units of carbon using a scaling factor of 0.47, following CARB's guidance (Gonzalez et al., 2015) and allowing us to match the carbon units in the offset project documentation. The LEMMA product also provides biomass by individual tree species at 30 m for a single year, 2012, which allowed us to compare the species composition of different areas.

To quantify the harvest history in offset projects and in other areas used as controls, we used a Landsat‐derived record of disturbance for California from 1985 to 2021 (Wang et al., 2022). Due to challenges in detecting disturbances at the beginning of the time series, we omit estimates from the year 1985 and analyze disturbances from 1986 to 2021, aligning with the start year of the eMapR and LEMMA biomass datasets. This disturbance dataset uses the Continuous Change Detection and Classification algorithm (Zhu & Woodcock, 2014) to identify abrupt changes in land surface characteristics across California based on time‐series surface reflectance at each 30 m × 30 m pixel from Collection 2 Landsat imagery (Masek et al., 2020). These changes are then attributed to disturbance causes (fire, harvest, or die‐off) using a random forest model trained on archival geospatial datasets of disturbance. For this study, we extracted the harvest component specifically, giving us a record of where forest harvest occurred each year over 1986–2021. These data provide a binary layer of harvest/no‐harvest for each pixel but do not quantify harvest intensity. We present “harvest rate” as a percentage representing the fraction of area harvested per year in a given region of interest.

We systematically evaluated the three datasets for the purposes of this study, that is, to track relative changes in carbon and harvest across the landscape for different regions of interest. We quantitatively compared eMapR and LEMMA against project‐reported carbon stocks and trends. We also visualized eMapR carbon, LEMMA carbon, and harvest changes for one example project with high rates of disturbance, CAR1066, to qualitatively assess agreement over the time period of 1986–2017 when all three datasets are available. For all relevant figures, we show results based on both eMapR and LEMMA. Due to a lack of eMapR and LEMMA data development beyond 2017, we were restricted to this smaller time window and could not address our carbon hypotheses (#1, 4) as robustly as the harvest hypotheses (#2, 5) for which data are available through 2021.

#### 2.1.3 Land ownership

In several of our analyses, we compared offset project lands to other privately owned forestlands in California by excluding public lands labeled by the California Department of Forestry and Fire Protection. We obtained these public lands data from the California State Geoportal (https://gis.data.ca.gov/datasets/f73858e200634ca888b19ca8c78e3aed_0/explore, accessed September 1, 2021). For other analyses, we compared specific timber companies' offset project lands against their other land holdings, using private land ownership data provided by the CalLands database (Macaulay & Butsic, 2017), available at https://callands.ucanr.edu/.

### 2.2 Comparison of carbon stocks and accumulation rates

In the first stage of our analysis, we explored both eMapR and LEMMA records of aboveground forest biomass as largely independent sources to corroborate the carbon stocks and trends reported in the offset project documentation. We used Google Earth Engine (Gorelick et al., 2017) to extract and average eMapR and LEMMA data for each project polygon over the same period that each project has reported carbon stocks (up to 2017, after which eMapR and LEMMA data are not available). We then plotted time series comparing the three datasets and calculated mean stocks and trends. For carbon stocks, carbon trends, and mean harvest rates, we report metrics by project as well as a mean and standard error across the 37 projects, weighted by the area of each project. We also provide validation of the three datasets in the Supporting Information, comparing eMapR versus LEMMA stocks and trends against project documentation, and assessing qualitative agreement in relative changes between eMapR, LEMMA, and harvest for an example project, CAR1066.

To gain insight into the incentives and long‐term strategies of these carbon offset projects, we calculated the ratio of credits earned at the beginning of the project from the initial stock above baseline to the credits earned during the project from incremental carbon accumulation (Figure 1). This ratio allowed us to identify the dominant source of crediting to date and to estimate the amount of time required for crediting from incremental carbon accumulation to exceed the initial payout.

### 2.3 Spatiotemporal comparison of projects to similar lands

As a method of estimating the additionality of carbon in offset project lands, we compared time series of carbon and harvest in offset project lands to time series of carbon and harvest in similar privately owned forestlands. We used three different methods to delineate similar but non‐offset lands, representing alternative business‐as‐usual scenarios or approximate control groups. These control groups allowed us to infer the presence of additionality along the hypotheses presented in Table 1, that is, whether carbon sequestration and harvest in the offset projects were different from what they would otherwise be.

In the first method of defining an approximate spatial control group, we drew a 2 km surrounding buffer region around each project, excluding urban or agricultural lands as defined by the National Land Cover Database for 2016 (Homer et al., 2020) and publicly owned lands as defined by the California Department of Forestry and Fire Protection. This 2 km surrounding region represents a land area similar in size to most projects. The approach has been used by previous forestry studies (e.g., Yang et al., 2021) to design controls in a systematic way, with geographic adjacency between test (i.e., project) and control regions ensuring that environmental, climate, and ecological conditions are on average likely to be similar.

In the second method, we defined larger (regional) control groups: all private forestlands in either the “coastal region” or “interior region” of northern California. The coastal region consists of the Northern California Coast supersection plus the western part of the Southern Cascades supersection (USFS ecosections 263A and M261B). The interior region consists of the eastern Southern Cascades (excluding M261B), Modoc Plateau, and Sierra Nevada supersections north of 39.7° N. We found it appropriate to consider these two regions separately given their substantial ecological differences and diverging patterns of carbon and harvest over time.

In the third method (presented in the Supporting Information), we followed the approach of several previous studies using covariate matching to identify control groups for each project (e.g., Andam et al., 2008; Ferraro et al., 2011; Stuart, 2010). We used three covariates: PRISM mean annual temperature and precipitation normals for 1990–2020 (Daly et al., 2008) and “site productivity class,” a metric for forest productivity provided by the USFS from FIA data (obtained from B. Wilson, cited in Tubbesing et al., 2020). For this approach, all data were regridded to 800 m to match the PRISM climate data. Then, we calculated the Mahalanobis distance between each project mean and all other pixels of the same region (coastal or interior) in the three‐dimensional standardized space of temperature, precipitation, and site class. Each project's “control” carbon and harvest time series consisted of the average of the most similar *n* number of pixels, where *n* is chosen for each project to approximate same area as the project (mean = 53 pixels, ranging from 7 to 225).

Finally, we also provide case studies quantifying differences in carbon accumulation, harvest, and species composition for two large timber companies' offset versus non‐offset land holdings. Sierra Pacific Industries (SPI) is one of the largest timber companies and landowners in the United States and has submitted approximately 30.5% of its California land area for active or proposed IFM projects. Green Diamond Resource Company owns primarily coastal redwood timberlands and owns one active IFM project in California, CAR1339, representing 11.9% of its land holdings in California. We investigated whether active or proposed offset lands have statistically distinct amounts of carbon or harvest compared to other lands by each owner. Preferential selection of lands that have most recently been harvested, for example, could allow the company to continue harvesting as business‐as‐usual on other lands while earning credits for lands it has recently harvested, profited from, and is now waiting to regenerate regardless of the offsets program.

#### 2.3.1 Hypotheses 1 and 2: Assessing pre‐project carbon accumulation and harvest

We first considered the available historical record of carbon and harvest leading up to the offset program initiation (1986–2012), comparing carbon stocks, carbon accumulation, and harvest rates for project areas versus control areas. We report the mean quantities and standard error over the 27 years. For harvest rates, which are highly variable year‐to‐year, we also performed a paired (relative) *t*‐test across years to assess whether the projects' harvest rates are consistently above or below those of the control areas.

#### 2.3.2 Hypothesis 3: Assessing pre‐project species composition

Next, we investigated the species composition of projects versus the spatial controls, using the species‐level biomass data provided by LEMMA. This allowed us to quantify whether project areas had a higher or lower density of particularly valuable timber species like redwood and Douglas‐fir prior or at the time of earliest project initiation in 2012. For an analysis of redwood composition, we focused on the Northern California Coast supersection (USFS ecosection 263A, green in Figure 2) which is characterized by redwood stands. We performed paired *t*‐tests comparing the density of a given species in each project to its density in the projects' surroundings. This tree species comparison allowed us to estimate whether there is otherwise high demand for harvest in the projects. Because the species data were only available for 2012, we were not able to compare species composition longitudinally or for before‐vs‐after project initiation in this study; hence, there is no Hypothesis 6 for post‐project changes in species composition.

#### 2.3.3 Hypotheses 4 and 5: Assessing post‐project change in carbon accumulation and harvest

We then quantified how much carbon accumulation and harvest was occurring in offset project lands compared to the spatial controls, before and after different projects were initiated. For these before‐and‐after comparisons, we only considered the 16 projects which started by 2014; this allowed for at least three points of eMapR or LEMMA data (2015–2017), and seven points of harvest data (2015–2021) after project initiation. These 16 projects accounted for 37% of all project area and 41% of credits issued to date from the full set of 37 projects. We calculated after‐minus‐before changes, testing for statistical significance using a Chow test on differences in carbon slope and a paired *t‐*test on differences between average harvest rates for each project.

## 3 RESULTS

### 3.1 Comparison of carbon stocks and accumulation rates

Across the 37 IFM projects in California, we found that eMapR and LEMMA records of average carbon stocks varied from those reported in project documentation (root mean square error = 30.9 and 29.2 ton C/ha, respectively; Figure S1). However, the remote sensing products did not show a clear bias in terms of consistent over‐ or underestimation relative to project‐reported carbon stocks for individual projects. One exception was for projects with high reported carbon densities, where we did find a slight underestimation, as expected.

**FIGURE 3:**
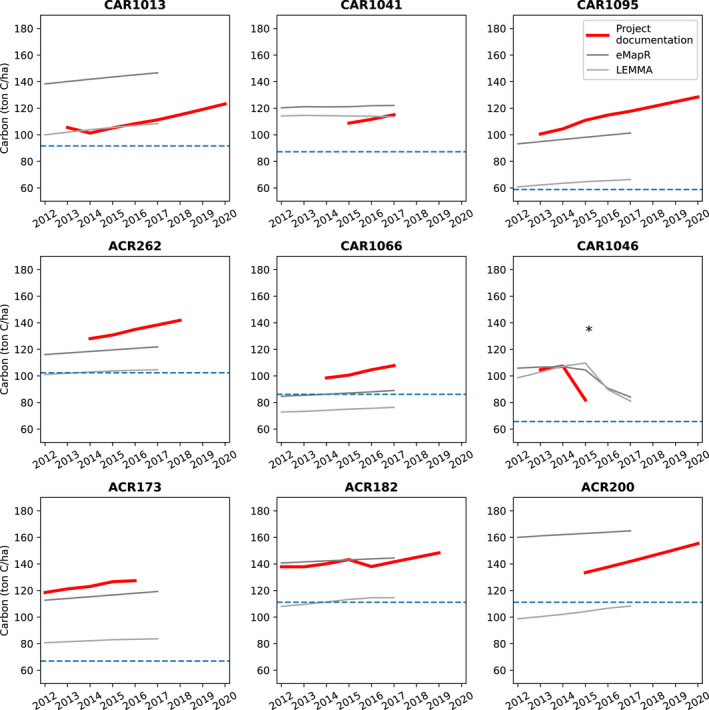
Three datasets of carbon stocks in improved forest management (IFM) projects. Here we show comparisons of three different data sources for a sample of the nine largest projects that started between 2013 and 2015 (labeled in Figure 2). Baseline carbon stocks as reported in project documentation are indicated by horizontal dashed lines. Remote sensing‐derived estimates of aboveground carbon stocks (gray) show slower rates of carbon accumulation than those reported in project documentation (red), with approximately half of the total carbon accumulation over time. The asterisk (*) in CAR1046 indicates the Route Complex Fire in 2015 which ultimately led to the project being terminated.

For carbon accumulation rates, the remote sensing‐derived estimates were considerably different from the project‐reported inventories. Specifically, projects reported 2.4 times higher rates of carbon accumulation than eMapR or LEMMA, with the average project‐reported rate (weighted by project area) being 1.97 ± 0.54 ton C/ha/year versus 0.83 ± 0.16 ton C/ha/year for eMapR and 0.82 ± 0.22 for LEMMA (see Table S2 for full details by project). Here error is reported as standard error across the sample of 37 projects. Projects' rates of carbon accumulation were variable and likely dependent on stand age, with some as high as 4%–5% per year averaged over the past 4–6 years according to project documentation. The carbon time series for nine of the largest and longest running offset projects highlights the discrepancy in carbon accumulation rate among data sources (Figure 3). As described in Section 2, the Landsat‐derived estimates of carbon accumulation used here may have a low bias at high AGL carbon (and high leaf area), contributing in part to the difference with the project documentation; further quantitative assessment of potential absolute differences may require next‐generation remote sensing products that are currently in development, leveraging new observations from Global Ecosystem Dynamics Investigation (GEDI), and other lidar products (Dubayah et al., 2020). However, despite the differences between eMapR, LEMMA, and project‐reported carbon, the eMapR and LEMMA products show relatively high levels of agreement in being able to track relative changes associated with harvest disturbance patches at a landscape scale (Figure S2).

Excluding CAR1046 (terminated due to fire) and seven other projects that began as early action projects prior to 2012, the initial credits issued to IFM projects in California were on average 26.5 ± 5.9 times greater than the number of credits issued annually thereafter. Thus, we can expect that if current forest growth rates continue, it would take 26.5 years on average for the incremental growth to become the dominant source of payment. Since this estimate is comparable to the project crediting period of 25 years, we expect that the subsequent trajectory of carbon accumulation may serve an important (non‐negligible) revenue stream for many projects, and potentially a dominant term for several projects. This variation in growth rate versus initial stocks is demonstrated in Figure S3. Large timber companies (i.e., SPI, Green Diamond Resource Company, and Mendocino Redwood Company) are more likely to have high growth rates but lower initial stocks, with an average of 23.1 ton C/ha above the baseline compared to 39.2 ton C/ha above the baseline for other projects. The break‐even times for accumulation credits equaling initial above‐baseline credits varied from 3 to 93 years for different projects.

### 3.2 Spatiotemporal comparison of projects to similar lands

**FIGURE 4:**
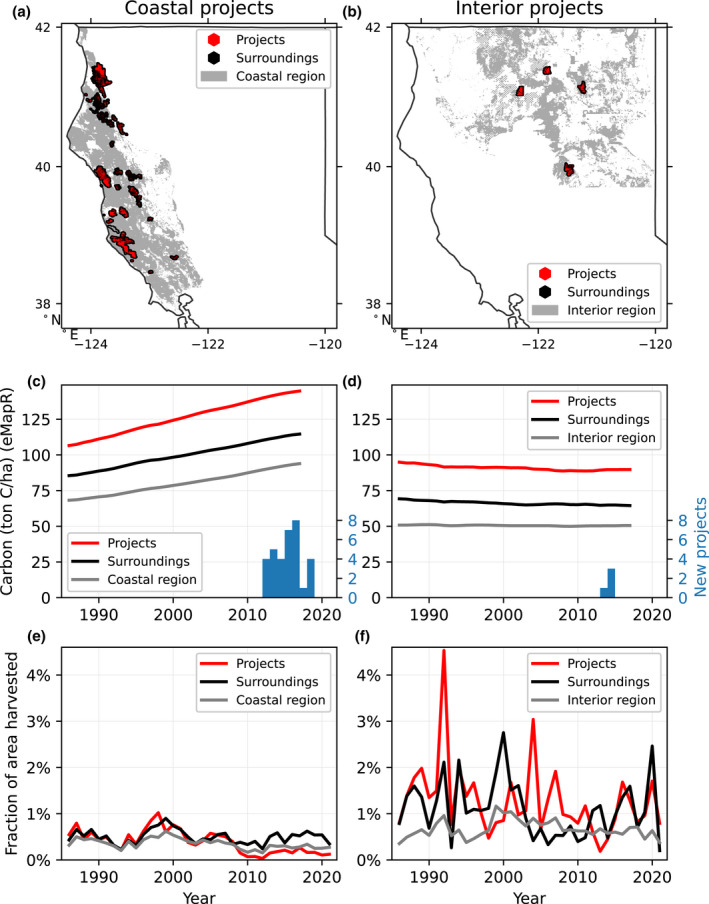
Carbon and harvest trends in offset projects and surrounding lands. We divided projects into two groups: 33 projects in the “coastal region” of Northern California Coast plus western Southern Cascades (a, c, e) and four projects in the “interior region” of the eastern Southern Cascades, Modoc Plateau, and Northern Sierra Nevada (b, d, f). We then compared carbon (eMapR) and harvest data for the combined offset project lands (red), a 2 km surrounding area of private forests around offset projects (black), and all private forests of the broader region (gray). Offset project lands follow carbon trajectories similar to other forests, both before and after projects begin (c, d). Offset project lands have historically been harvested more intensely than surrounding lands, especially in the interior region (e, f). Figure S4 includes similar patterns for LEMMA carbon and the third system of matched controls.

Using carbon data from eMapR and LEMMA, and harvest data from Wang et al. (2022), we quantified differences in the time series for offset project lands compared to three control groups: a 2 km surrounding region around projects, a broader region of either coastal or interior northern California (Figure 4a,b), and a set of covariate‐matched 800 m pixels. We found broadly consistent patterns of carbon accumulation between eMapR and LEMMA and between the “surrounding” and “covariate‐matched” controls. Therefore, for conciseness in the main text, we focus our primary analysis on eMapR and the first two systems of controls, with results for LEMMA and the matched controls system presented in the Supporting Information (Figures S4–S6).

#### 3.2.1 Hypotheses 1 and 2: Pre‐project carbon accumulation and harvest

Most offset projects, located in the coastal region, have relatively high carbon stocks and have been accumulating carbon over the past three decades, both before and after the offset program began (Figure 4c). Over the pre‐project period of 1986–2012, project areas had consistently higher carbon stocks than control groups. For coastal projects, the mean carbon stock was 123.0 ± 1.9 ton C/ha, which was higher than the surrounding areas (97.5 ± 1.4) and coastal region (78.0 ± 1.2). Similarly, for interior projects, the mean carbon stock was 91.2 ± 0.3 ton C/ha, which was higher than the surrounding areas (66.5 ± 0.2) and interior region (50.6 ± 0.06). Here the reported errors represent standard error in stocks over the 27‐year record.

The pre‐project carbon accumulation rate for the combined projects area was different (in absolute units) than the rate in nearby forests (1.30 ton C/ha/year for coastal projects vs. 0.95 for surroundings or 0.82 for coastal region; −0.22 for interior projects vs. −0.15 for surroundings or −0.04 for interior region). However, as a percent change, all three coastal areas—that is, projects, surroundings, and the full region—were growing at 1.0%–1.1% per year, and all three interior areas showed negligible (≤0.2%) change per year. Therefore, as a percent change, the total carbon added in projects beyond what would be expected based on these regional average rates of accumulation is effectively zero. The finding of consistently increasing carbon stocks across the coastal region is at odds with Hypothesis 1 regarding the static baseline for carbon.

For harvest, we found a general pattern of decline since the early 2000s, and an especially steep decline starting in 2008, a few years before any projects began (Figure 4e,f). Project areas had mostly higher harvest rates (measured as the fraction of area harvested) than their immediate surroundings prior to 2012, particularly for the interior region. Over the period of 1986–2012 preceding the offsets program, the combined coastal project areas were harvested at about the same rate as their surroundings (harvest rate differences were not statistically significant) and 17% more relative to the broader coastal region (paired *t*‐test across years, *p* = .004). The combined interior project areas were harvested 69% more, relative to their surroundings (*p* = .12, not statistically significant), and 106% more than (more than twice as much as) the broader interior region (*p* < .001). These four interior projects with particularly high harvest are owned by SPI, a large timber company. Looking across SPI lands, we found that areas of active or proposed offset projects were harvested 27% more than the rest of its properties in California during the same period of 1986–2012 (*p* < .001), and 31% more during 2008–2012 (*p* = .002). This finding of disproportionate rates of historical harvest on project lands is at odds with Hypothesis 2 regarding recovery from harvest.

#### 3.2.2 Hypothesis 3: Pre‐project species composition

**FIGURE 5:**
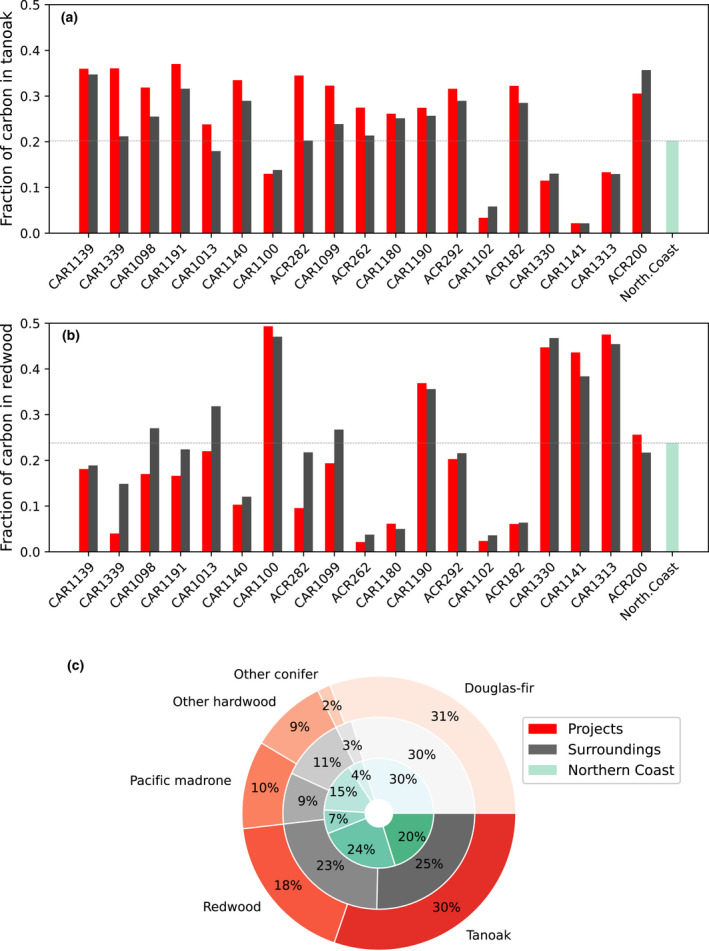
Species composition of Northern Coast offset projects. Offset projects in the Northern Coast supersection (Figure 2) have significantly more of their carbon as tanoak (a, bottom right of c) and less as redwood (b, bottom left of c), compared to surrounding non‐offset areas in 2012. This is particularly true for timber company‐owned projects (CAR1339, CAR1191, CAR1190). This suggests that harvest value in project lands was lower than surrounding areas prior to projects' start, and credits issued to many of these projects may not actually be preventing greater harvest.

**FIGURE 6:**
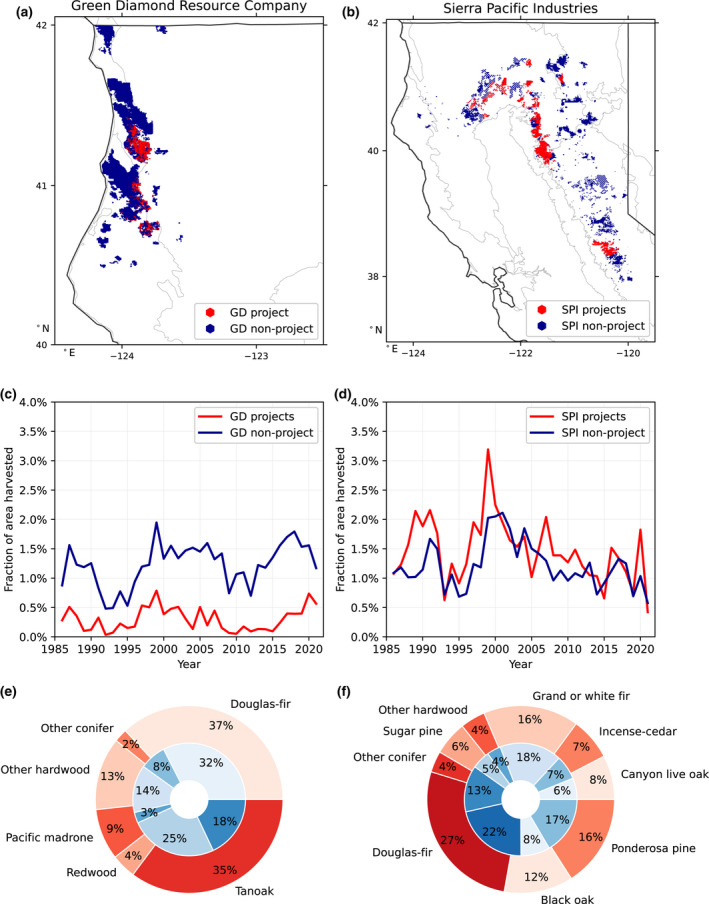
Divergent strategies of offset project selection between two large timber companies. Green Diamond Resource Company (GD, left) and Sierra Pacific Industries (SPI, right) demonstrate different strategies in the selection of their lands for proposed or active offset projects (red) versus the rest of their land holdings (blue). GD lands are located predominantly in the Northern California Coast (a). GD project lands (currently one project, CAR1339) have a very intricate delineation, around areas that have been historically harvested *less* (c), and have nearly double the fraction of tanoak (a less timber‐valuable species) and only one‐sixth the redwood of their other properties (e). SPI lands, on the other hand, are predominantly in the fir and pine forests of interior California (b, f), and their offset project lands have been harvested 26% *more* than their other properties over 1986–2012 (d).

Next, we focused on the Northern California Coast supersection for an analysis of tree species composition in offset areas compared to other areas. Much of this region is coast redwood forest which has high harvest value, mixed with tanoak which is a less valuable understory species (Waring & O'Hara, 2008). Using the LEMMA record of species composition available for 2012, at the time that the offset program began, we found statistically significantly higher tanoak density in offset project stands (30.3%) compared to their immediate surroundings (25.4%) or the supersection mean (20.2%) (*p* < .001 for both paired *t*‐tests; Figure 5). The discrepancy was higher for timber company‐owned projects, which had 34.7% tanoak compared to their immediate surroundings with 26.1% tanoak. For the Green Diamond Resource Company specifically, their IFM (CAR1339) is drawn around stands with particularly high tanoak density (35.2%) and low redwood (4.3%), versus the rest of its properties which are 17.9% tanoak and 25.2% redwood by carbon density (Figure 6). The project documentation for CAR1339 is consistent with this finding from the LEMMA data, reporting that tanoak constitutes more than half the basal area included in the IFM. In this case study, the Green Diamond project lands were also historically harvested less than their other properties, in contrast to the SPI projects discussed previously which were historically harvested more. This finding of projects being drawn around less valuable stands is at odds with Hypothesis #3 regarding projects protecting forests otherwise at risk of harvest.

#### 3.2.3 Hypotheses 4 and 5: Post‐project change in carbon accumulation and harvest

Finally, we compared carbon accumulation and harvest rates for each project over equal time periods before and after project initiation for the 16 projects that started by 2014 (Figure S5). Regarding carbon, 12 projects showed a decrease in eMapR carbon accumulation rate after initiation, 10 of which were statistically significant as measured by a Chow test with *p* < .05. Two other projects not included here, CAR1046 (terminated) and CAR1174, have also lost significant amounts of carbon due to fires in 2015 and 2018. The other four projects showed insignificant increases in carbon accumulation rate. One project, CAR1092, showed a significant increase in harvest rate after initiation.

**FIGURE 7:**
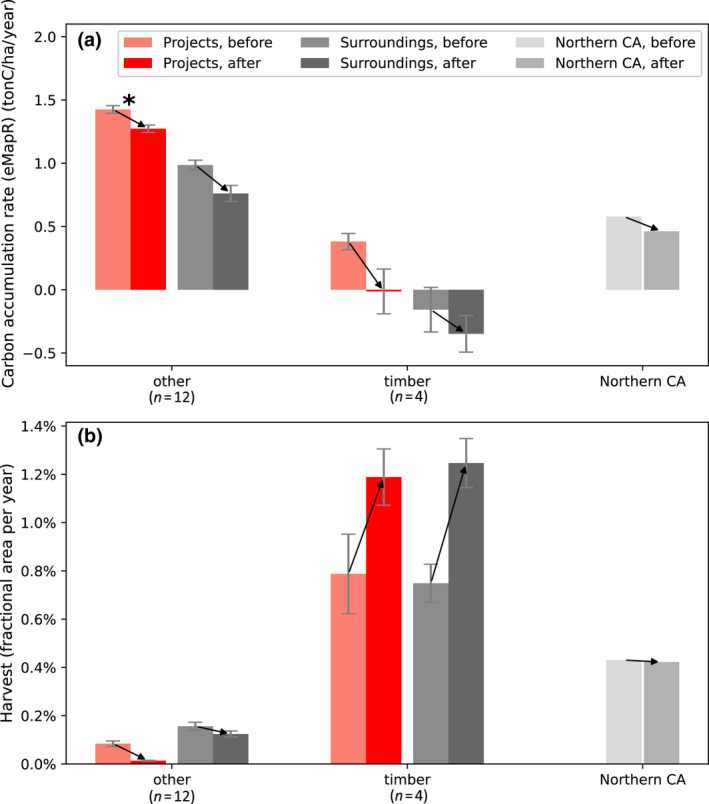
Carbon and harvest changes by landowner category. Offset projects are owned by a variety of conservation groups, individuals, timber companies, other for‐profit companies, and tribes. Carbon accumulation rate has declined broadly across Northern CA, including in offset projects and nearby areas regardless of landowner type (a). (b) Harvesting rates across Northern CA have remained fairly constant, with no indication of harvest reductions in offset projects. In fact, we observe a slight increase in harvest on large timber company‐owned offset projects and their surroundings. CAR1046 was excluded since it burned in 2015 and was terminated. Statistical significance in a paired *t*‐test is indicated by an asterisk (*); most changes are not statistically significant largely due to small sample size and low power.

We also compared before‐and‐after rates of carbon accumulation and harvest for these 16 projects grouped into two landowner categories: large timber companies (SPI) and others (Figure 7; landowner information provided in Table S3). This analysis revealed that carbon accumulation rates have been decreasing across Northern California forests, including offset project lands, which show a statistically significant decline in accumulation rate since initiation (*p* < .05) according to eMapR. Harvest rates have increased slightly (not statistically significant) on the large timber companies' offset project lands as well as their surroundings, and have decreased slightly in the combined 12 other projects. These findings of a general lack of increase in carbon accumulation or significant decrease in harvest are at odds with Hypotheses 4 (carbon) and 5 (harvest) regarding the expected changes after project initiation.

## 4 DISCUSSION

In this study, we applied three geospatial remote sensing‐based data products to systematically assess carbon and harvest in IFM compliance offset projects within California. Our comparison between eMapR, LEMMA, and project‐reported carbon revealed several differences, including projects reporting considerably faster accumulation of carbon than estimates derived from eMapR or LEMMA. Per the IFM protocol, projects may rely on approved forest growth models (which do not incorporate climate impacts on growth projections) to report carbon stocks for up to 12 years at a time, and may adjust those estimates before receiving credits, for which ground‐based inventories are required. Our finding about the rate of carbon accumulation contributing a similar number of credits after approximately 26.5 years as those initially awarded for above‐baseline stocking suggests that both high initial stocks and high growth rates (perhaps due to recovery from previous harvest and other edaphic factors that accelerate growth) are in the projects' financial interest under existing protocol. For large timber company lands which have historically harvested more intensely and start with a lower initial stock, the greater incentive is for landowners to place offset projects on lands with the greatest potential for sustained growth rather than protecting carbon stocks in already‐dense stands.

By comparing carbon and harvest trends in offset project lands to other similar lands, we can infer the extent to which carbon that has accumulated is truly additional to what may have accumulated otherwise. We found five lines of evidence which cast substantial doubt on additionality (project‐by‐project breakdown in Table S4), with carbon stocks and accumulation that very likely would have occurred regardless of the offsets program.

First, regarding Hypothesis 1, the fact that eMapR and LEMMA show a long‐term accumulation over 1986–2017 for all coastal regions suggests that the existing protocol which always draws flat baselines may not be realistic for many California forests; the real‐world baseline in this case would be a slow increase. In other words, the accumulation in these project lands may be attributed to this broader trend for private forestlands recovering from high levels of harvest in the 1950s–1970s (Morgan et al., 2004) and is not necessarily a consequence of specific management on project lands. Although these projects have carbon stocks above baseline levels, the fact that project relative carbon accumulation rates track the rates observed in control regions suggests that credited incremental growth may not be additional. The widespread positive rates of accumulation also weaken the protocol's assumption that offset stocks would otherwise be reduced to baseline levels. This potential over‐crediting adds to over‐crediting concerns from previous research related to how baselines are defined (Badgley et al., 2021).

Regarding Hypothesis 2, we found that many project areas have been harvested more than other areas over the historical period, especially for large timber companies such as SPI in the interior region, and may now be receiving credit for the natural recovery of those forests. Longer‐term monitoring would be required to quantify whether these areas will recover and stay recovered beyond the remainder of the expected rotation periods.

Regarding Hypothesis 3, the disproportionately high tanoak density in Northern Coast IFM projects (such as the Green Diamond Resource Company project) suggests that these lands have lower harvest value than nearby private forestlands and are therefore at lower risk of logging. Although theoretically landowners could replace the tanoak with redwood seedlings, the lack of harvest on these tanoak‐dense stands such as CAR1339 (Figure 6) over recent decades suggests little intention of timber production such that protecting these areas as offsets would offer limited additional climate benefit. Protecting disproportionately tanoak stands is likely also not in the best interest of maximizing carbon storage, as the species is much less carbon dense per area than conifers like redwood.

Regarding Hypothesis 4, none of the projects across our subset of the 16 longest‐running projects demonstrated a statistically significant increase in their eMapR carbon accumulation rate after initiation (one project showed a significant increase according to LEMMA). Instead, most projects demonstrated decreases, following a similar pattern as the controls. While protocol rules do not require projects to increase their carbon accumulation rates, as projects can claim to avoid baseline scenarios that significantly degrade carbon stocks instead, the general lack of increases among projects in our sample was striking. We would expect that IFM practices such as increasing rotation length would lead to increased carbon sequestration (due to lack of active carbon removal), observable in the first few years. However, a longer observational record may be required. Decreases in carbon accumulation in the past several years were also observed in non‐project lands and coincide with increased disturbances like drought, fire, and the sudden oak death pathogen which could threaten project permanence over the full duration of the projects' lifetime. Considering the findings from Hypothesis 4 alongside Hypothesis 1 demonstrates how the current system of defining static baselines fails to accurately capture both the expected carbon growth over time and the projects' performance (either to add carbon or prevent degradation) relative to what might otherwise occur.

Regarding Hypothesis 5, we found no evidence that timber companies are substantially reducing their harvest activity on offset project lands. In fact, we found some indication that harvest *may* be increasing, and carbon accumulation decreasing, in timber company projects and surrounding lands. The offset protocol credits initial stocks above the baseline and considers incremental growth additional, even when the harvest rate increases slightly and carbon accumulation rate decreases but remains positive. However, these inferences are based on only 3–5 years of post‐project carbon data and 7–9 years of post‐project harvest data, so longer monitoring is required to more confidently assess additionality by this method. Longer monitoring would also be needed to detect the carbon impacts of extending rotation length in timberlands. Another caveat for timber projects is that the protocol also provides credits for carbon in harvested wood products, which may allow some of the increased harvest activity to still be related to additional and permanent carbon storage (a topic beyond the scope of this study). In general, though, our finding that in most cases, landowners are able to both continue harvesting at previous rates and receive carbon credits suggests that the current protocol may be over‐crediting for naturally productive stands.

We acknowledge some well‐known uncertainties and limitations in the remote sensing observations, which differ from the uncertainties and limitations in the inventory or modeling approaches used to document a project's stocks. First, eMapR and LEMMA may not accurately capture incremental growth in closed‐canopy forests. Both tend to underestimate biomass at high densities (e.g., in the redwood forests, particularly with LEMMA), and eMapR calibration only included a small portion of northern California; however calibration did include diverse conifer‐dense stands (Battles et al., 2018; Kennedy et al., 2018). We therefore refrain from drawing any specific conclusions about the exact carbon stocks in project areas, but rather use these products to compare relative differences across the landscape, which are useful for evaluating additionality, and demonstrate the types of analyses that could benefit offset programs going forward. In general, we do not expect biases to impact the project areas differently than the control areas and therefore feel comfortable using them to capture signals of additionality and draw qualitative conclusions even if the exact magnitudes of change are uncertain. These products have also undergone peer review (Kennedy et al., 2018; Ohmann & Gregory, 2002), have been widely used for many carbon cycle applications at larger spatial scales (Bell et al., 2015, 2021; Zhou et al., 2021), and are demonstrated here to broadly capture the spatial structure of disturbance and post‐disturbance recovery (Figure S2). We also expect remote sensing products to continue improving for use at fine scales with IFM projects, with support by programs such as NASA's Carbon Monitoring System and new spaceborne lidar observations from GEDI (Dubayah et al., 2020). Second, the disturbance history dataset by Wang et al. (2022) was shown to have a 72% user's accuracy (omission) and 81% producer's accuracy (commission). Based on the nature of the disturbance detection, it is likely to be more accurate for capturing clear‐cut harvests as opposed to selective thinning. We may have underestimated the total amount of harvest but do not expect this to present a bias in comparing offset versus non‐offset lands. In general and importantly, though, the datasets are largely independent from the project data and enable larger‐scale analyses that would otherwise not be possible. The analyses therefore demonstrate the potential value of improved remote sensing observations for offset project verification.

Another caveat is that the spatial control groups we designed are imperfect estimates of a counterfactual scenario, which in reality is impossible to quantify precisely. It is theoretically possible that the offset lands would have otherwise diverged from the controls, for example by being harvested even more. In general, however, our approach for defining controls is systematic, reasonable, transparent, and we would expect these lands to face a similar risk of degradation. Our assessment is also thorough in exploring three distinct definitions of control groups which yield the same broad conclusions of lack of additionality. Although imperfect, remote sensing tools enable the design of effective controls that make it possible to characterize the counterfactual additionality claims made across the IFM project portfolio as a whole.

We intend this analysis to serve as a constructive criticism for the offsets program, which could benefit by incorporating more geospatial analyses of carbon and harvest trends. A next generation IFM protocol leveraging new remote sensing products and spatial controls could more accurately track additional climate benefits than the current system for defining baselines. By comparing observed trends in projects relative to similar “control” forests, an implicit baseline would be allowed to change over time, such as is the standard for some REDD+ projects and the Duke framework for forest offsets (Willey & Chameides, 2007). Such a system could require evidence of either carbon‐positive management or prevention of degradation, for example by documenting a divergence from historical trends or control areas, rather than a hypothetical counterfactual. It could also enable more accurate tracking of harvest risk based on species composition, particularly in coastal forests where harvest potential may vary as a consequence of degradation from previous harvest and land management. More tailored offset rules might involve weighing the benefits of potentially more permanent but less total carbon storage as tanoak as compared to redwood. Finally, a system based on comparison against controls could incentivize more holistic approaches to forest conservation, including resiliency against fire to help maintain carbon stocks that would otherwise decline. Such improvements to make the program more rigorous could help build confidence among credit issuing bodies, policymakers, and the public that climate targets are being met. Otherwise, we may be miscalculating net emissions while rewarding projects for little or no change in forest management. Improving California's crediting scheme could have a large global impact, with California serving as an example system for other offset programs nationally and internationally.

Our findings about declining rates of carbon accumulation and lack of evidence of additionality elucidate a need for more rigorous evaluation of carbon stocks, trends, and risks. The current protocol may be crediting projects on lands that are naturally productive or that feature low harvest potential, rather than inducing new climate mitigation outcomes. Our analyses also demonstrate an important role for geospatially complete, remote sensing‐based, and publicly available data products for monitoring carbon offset projects. These datasets for carbon and harvest allowed us to perform comprehensive comparisons of the trajectories of offset areas relative to other similar areas since the 1980s at a 30 m resolution. Such spatial and temporal completeness exceeds what is offered by plot‐level forest inventories (which are also often only privately accessible) or county‐level datasets. Completeness and public availability could also improve offset program buy‐in from smaller landowners and enable more large‐scale and transparent deployment of carbon offsetting. However, geospatial data products require continued scientific investment, validation, and annual updates to increase confidence in their accuracy for different forest types and over time.

## 5 CONCLUSION

We present a novel suite of analyses to (1) demonstrate the potential for remote sensing‐based data products in evaluating IFM offset projects and to (2) investigate the validity of additionality assumptions embedded in California's forest offset protocol. Although remote sensing‐based methods for estimating carbon stocks are not yet reliable replacements for on‐the‐ground measurements, they provide a reasonable and systematic basis for detecting changes between trends in lands enrolled in carbon offset projects and nearby control areas. In comparing carbon accumulation rates, harvest patterns, and species composition between project areas and similar private forestlands, we did not find evidence that IFM project carbon stocks are systematically at risk of being managed down to baseline levels, nor that carbon being added in IFM projects is additional to what might have been added in the absence of offset credit incentives. Implementing these types of analyses toward stricter standards in IFM protocol could both increase confidence in carbon additionality and enable the deployment of NCS at larger scales beyond the state of California.

## CONFLICT OF INTEREST

The authors declare no conflicts, financial or otherwise, that could be perceived as influencing the research described here. M. L. Goulden reports grants from the California Strategic Growth Council, and D. Cullenward is the Vice Chair of California's Independent Emissions Market Advisory Committee but does not speak for the Committee here.

## CODE AVAILABILITY STATEMENT

Python and Google Earth Engine code are provided in the repository as well as Github https://github.com/scoffiel/carbon_offsets/.

## Supporting information


Appendix S1

## Data Availability

All data come from publicly accessible sources described in Section 2.1 (Methods). We have compiled and packaged the specific CARB, eMapR, LEMMA, and harvest data we used into an online Dryad repository at https://doi.org/10.7280/D17D6W.
