# Supplementary material for: Using remote sensing to quantify the additional climate benefits of California forest carbon offset projects
Source: Glob Chang Biol. 2022 Sep 12;28(22):6789–806. doi: 10.1111/gcb.16380 (PMC9826164; doi:10.1111/gcb.16380)
Supplement: Supplementary file 1 — Appendix S1 [file GCB-28-6789-s001.pdf]

Supporting information

Using remote sensing to quantify the additional climate benefits of California forest carbon offset projects

Shane R. Coffield*, Cassandra D. Vo, Jonathan A. Wang, Grayson Badgley, Michael L. Goulden
Danny Cullenward, William R. L. Anderegg, James T. Randerson

scoffiel@uci.edu

Here we present 6 additional figures and 4 additional tables supporting the results presented in the main text.

Contents

Fig S1. Comparison of remote sensing and reported carbon stocks and trends
Fig S2. Demonstration of agreement across remote sensing datasets for an example project
Fig S3. Varying strategies of carbon crediting
Fig S4. Figure 4 with added results for LEMMA carbon timeseries
Fig S5. Before-and-after carbon accumulation and harvest rates by project
Fig S6. Figure 5 with added panel for LEMMA results and third control group
Table S1. Proportion of projects' reported AGL carbon to the total standing live pool
Table S2. Summary of carbon stocks and trends from three datasets
Table S3. Projects' landowner information
Table S4. Additionality criteria per project

Supporting Information

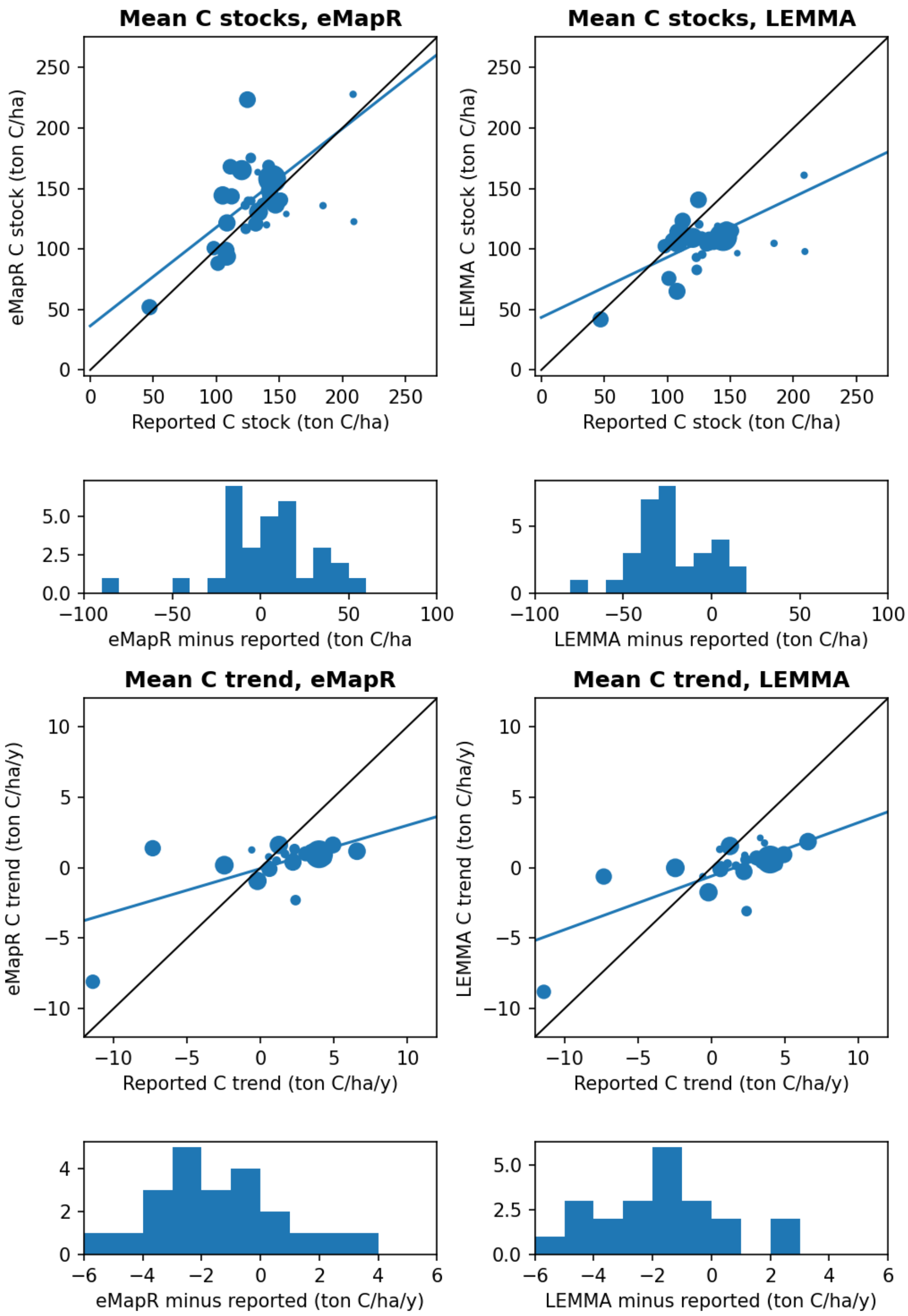

Fig S1. Comparison of remote sensing and reported carbon stocks and trends. eMapR and LEMMA estimates of carbon stocks are not significantly greater or less than reported carbon stocks across the portfolio of projects (top row), but have relatively high root mean square error against reported carbon stocks (30.9 and 29.2 ton C/ha, respectively). There is a slight bias to underestimate carbon stocks at high densities, especially with LEMMA. The largest discrepancies exist in estimations of the carbon accumulation rate over time (bottom half), with eMapR and LEMMA generally estimating lower magnitudes of change. Marker size indicates relative project area.

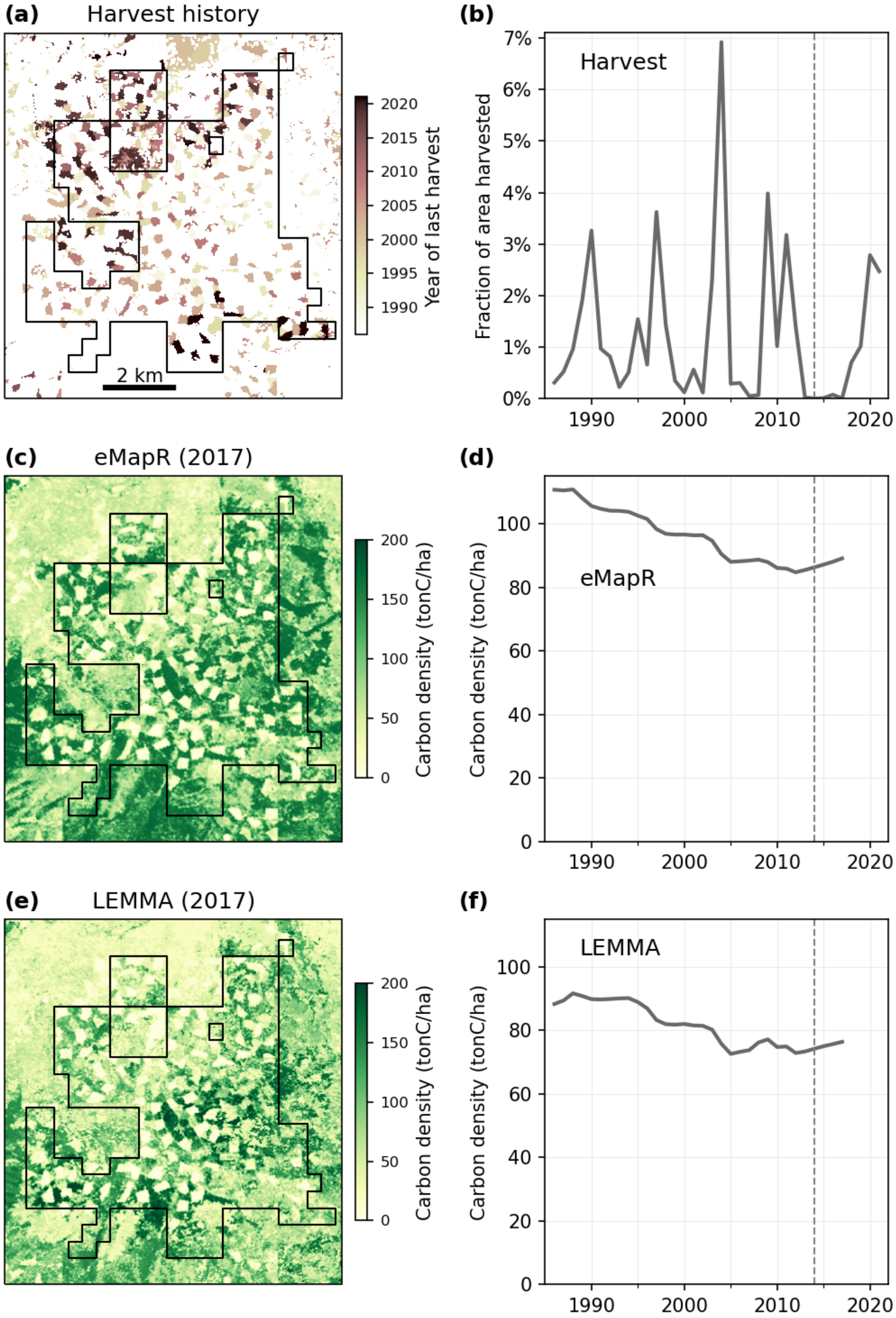

(a)
Harvest history
2 km
Year of last harvest
2020
2015
2010
2005
2000
1995
1990
(b)
Harvest
Fraction of area harvested
7%
6%
5%
4%
3%
2%
1%
0%
1990
2000
2010
2020
(c)
eMapR (2017)
Carbon density (tonC/ha)
200
150
100
50
0
(d)
eMapR
Carbon density (tonC/ha)
100
80
60
40
20
0
1990
2000
2010
2020
(e)
LEMMA (2017)
Carbon density (tonC/ha)
200
150
100
50
0
(f)
LEMMA
Carbon density (tonC/ha)
100
80
60
40
20
0
1990
2000
2010
2020

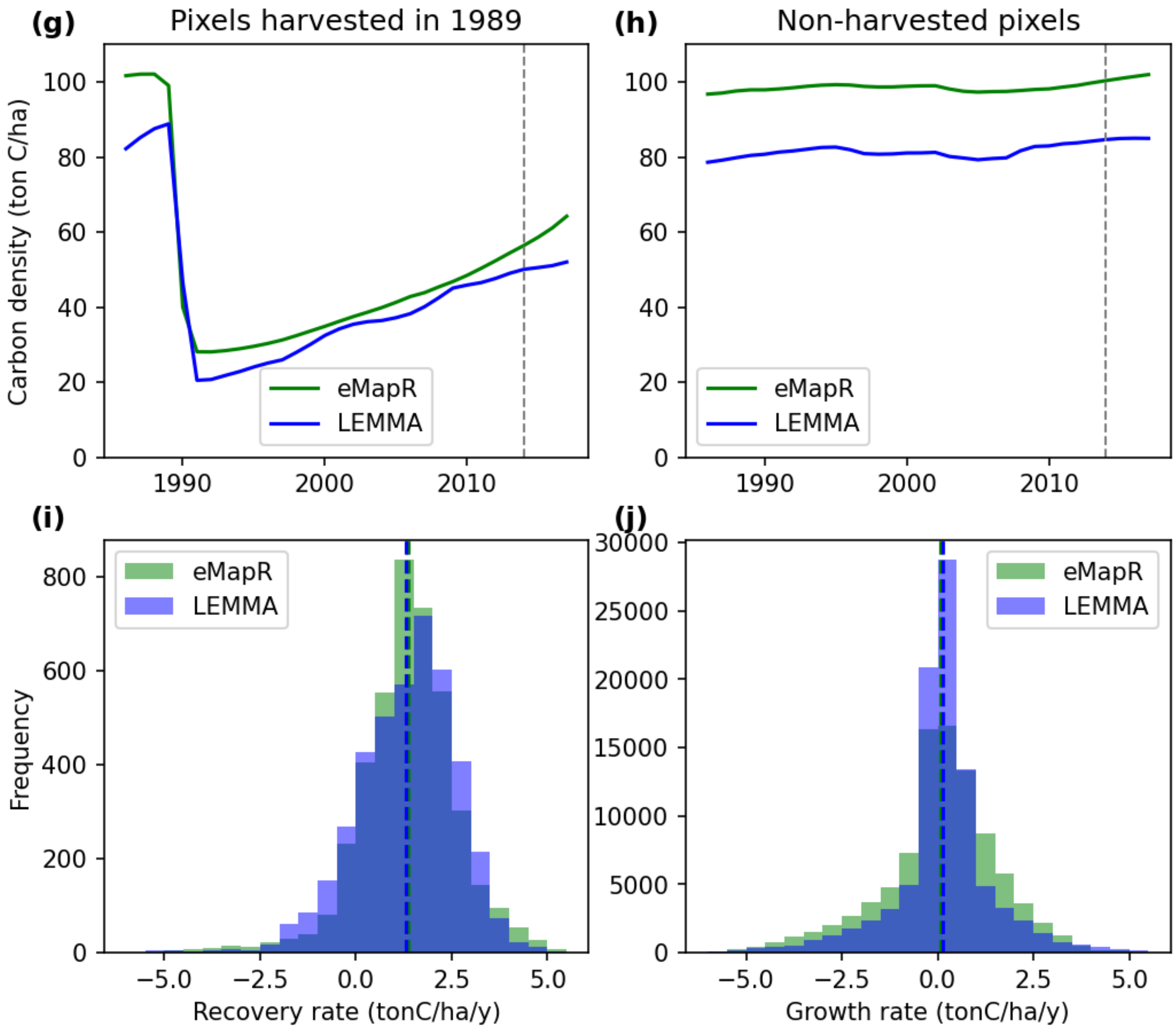

Fig S2. Demonstration of agreement across remote sensing datasets for an example project, CAR1066, which initiated in 2014 (dashed vertical line (b,d,f)). The harvest product by Wang et al. 2022 shows an expected pattern of patchwork clearcutting in this area over the available record of 1986-2021 (a). Harvest activity is fairly episodic, ranging from 0% to 7% of the area being cut each year (b). Both eMapR and LEMMA show substantial reductions in aboveground carbon in recently harvested patches (c, e), and loss of carbon in the timeseries aligns with large harvest events, despite the difference in absolute magnitude of carbon stocks between eMapR and LEMMA (d, f). Both eMapR and LEMMA capture steep declines followed by steady recovery for pixels that were harvested in 1989 (g) compared to non-harvested pixels over the record (h). The two carbon datasets agree in terms of rate of change of carbon over time, with approximately normal distributions of carbon sequestration rate across pixels, both for pixels recovering from harvest (i) or undisturbed (j). The means of the eMapR and LEMMA distributions are virtually equivalent as indicated by overlapping green and blue dashed lines.

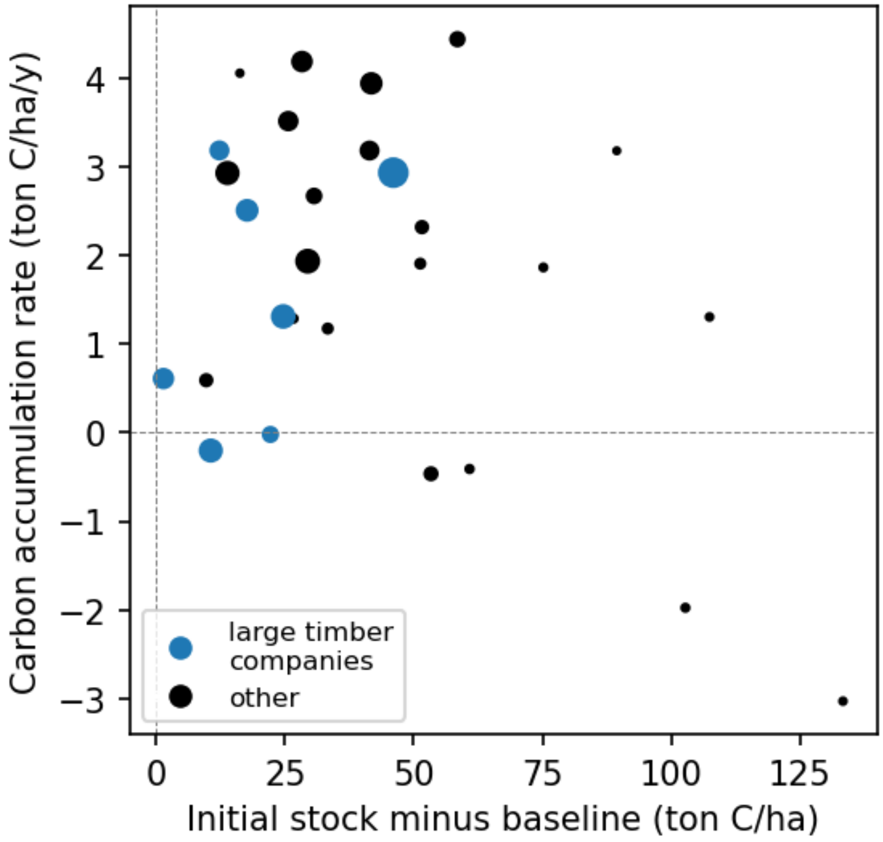

Figure S3. Varying strategies of carbon crediting. Projects receive credits for additional carbon stocks from (1) initial stocking above baseline levels and (2) subsequent incremental increases over time, minus any estimated leakage or secondary effects. This presents a trade-off; projects with lower initial stocks are more likely to have high rates of accumulation such that they are still feasible for substantial crediting long-term. This is particularly the case for projects owned by large timber companies (defined in Table S3). Marker size indicates relative project areas.

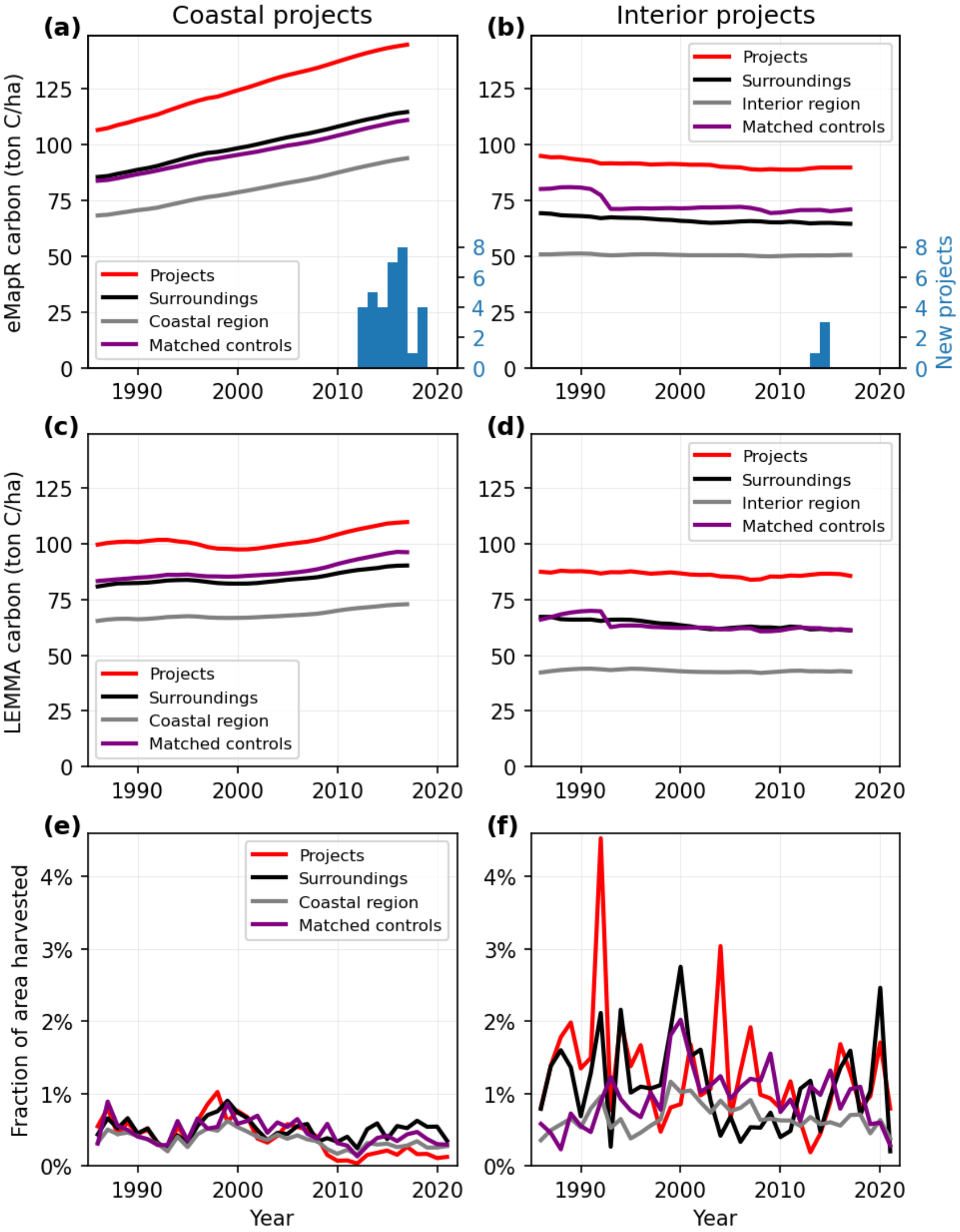

Fig S4. Copy of Fig. 4, with added results for LEMMA carbon timeseries (c, d) and a third system of spatial controls based on covariate matching (purple lines). The LEMMA carbon timeseries shows a slower growth of carbon in the coastal region (c), but similar patterns of projects being relatively carbon-dense and adding carbon at similar rates as the control groups. The third control group - based on Mahalanobis distance matching to other pixels with most similar temperature, precipitation, and productivity in the same region - is shown in purple. This system of controls gave qualitatively similar results to the “surroundings” in black, apart from a decline in carbon in 1993 in the interior controls which is attributable to the 1992 Fountain Fire.

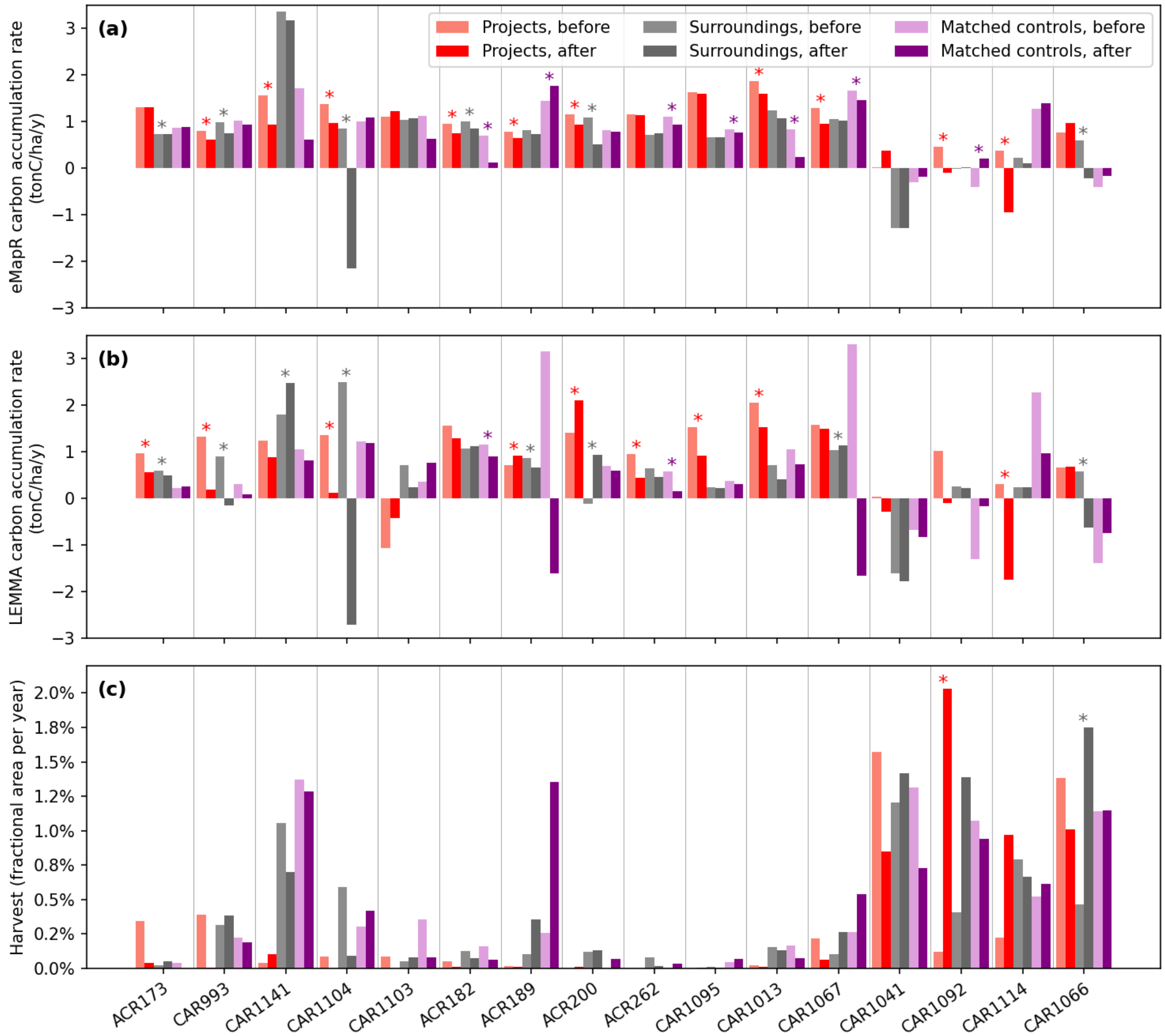

Fig S5: Before-and-after carbon accumulation and harvest rates by project. Asterisks* indicate statistical significance in the pre-to-post-project change, for an equal number of years considered before and after. For the 16 projects that started by 2014 (and excluding CAR1046 which terminated), 12 show a reduction in eMapR carbon accumulation rate after initiation (a), 10 of which are statistically significant (a). Four projects show an insignificant increase. LEMMA results are mostly similar, with predominantly decreases in carbon accumulation rate for projects (b); however one project, ACR200 does show a significant increase according to LEMMA. In terms of harvest, the four Sierra Pacific Industries projects (the four rightmost projects) show relatively high rates of harvest both before and after initiation. One project, CAR1092, has harvested significantly more since it became an offset project; none have harvested significantly less. The two systems of controls (gray and purple) differ in magnitudes of change but agree that the majority of areas show a decrease in carbon accumulation after projects begin.

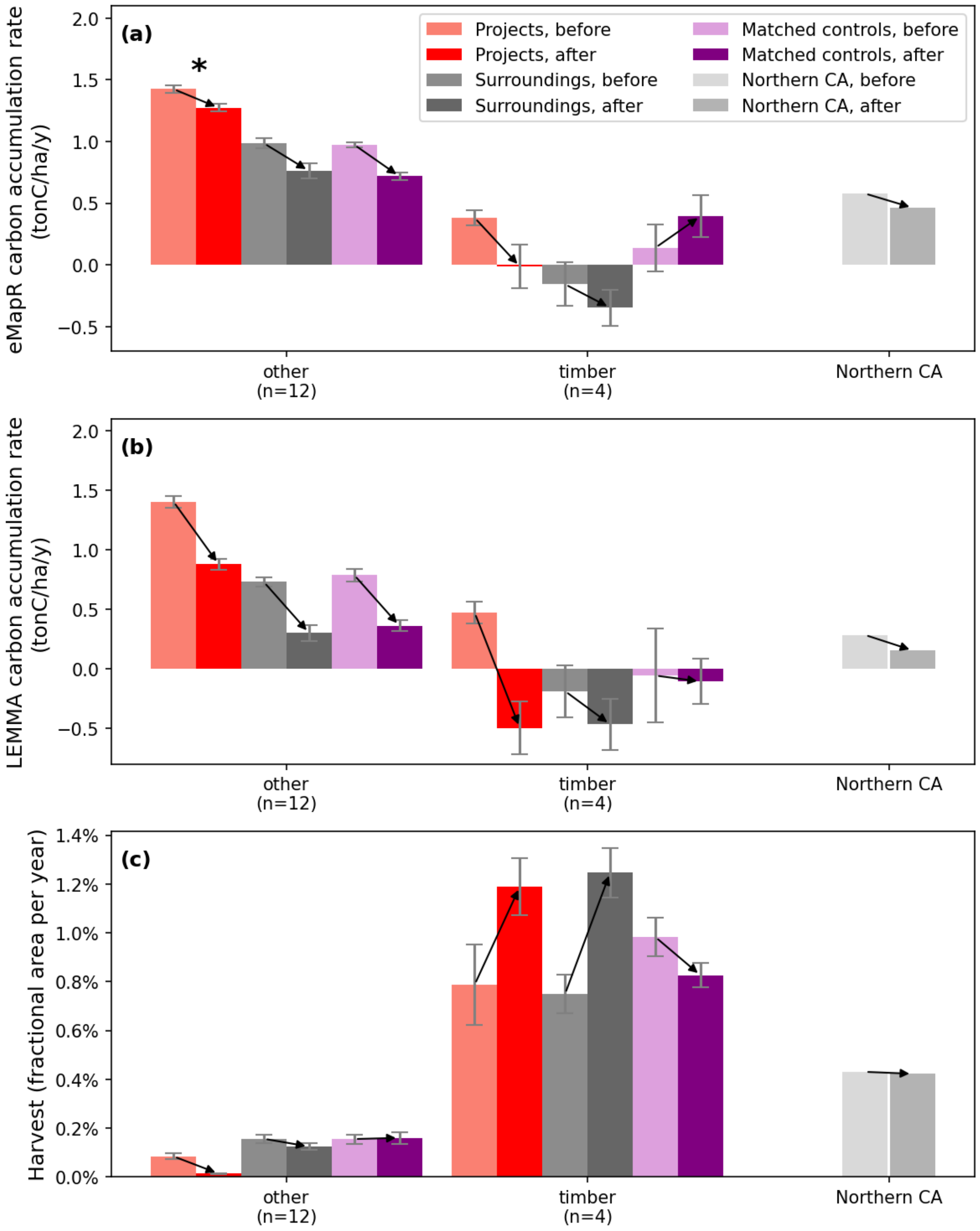

Fig S6: Copy of Fig. 5, with an added panel for LEMMA results (b) and added bars for the third system of controls (purple). At this scale of grouping projects into two landowner categories, eMapR and LEMMA show the same direction of change for all cases – i.e., a decline in carbon accumulation rate. The "matched controls" system performs similarly to the surrounding control areas, with the exception of the statistically insignificant increase in eMapR carbon accumulation and decrease in harvest rates for the timber company (interior) matched controls.

Table S1. Proportion of projects' reported above ground standing live carbon (AGL) to the total standing live pool.

Project ID	Average ratio of AGL to total carbon
ACR173	0.806
ACR182	0.809
ACR378	0.806
CAR1013	0.805
CAR1046	0.789
CAR1102	0.807
CAR1103	0.809
CAR1104	0.808
CAR1141	0.815
CAR1174	0.807
CAR1330	0.804
CAR1368	0.807
CAR993	0.808
**Average**	**0.806**
**Standard deviation**	**0.002**

Table S2. Summary of carbon stocks and trends from three datasets. For each project, stocks and accumulation rates are calculated over the time period for which both inventory and eMapR or LEMMA data are available, at most 2012-2017. Therefore projects starting in 2017 or later are reported as n/a.

Project ID	Area (ha)	Mean reported carbon stock (ton C/ha)	Mean eMapR carbon stock (ton C/ha)	Mean LEMMA carbon stock (ton C/ha)	Mean reported carbon accumulation (ton C/ha/y)	Mean eMapR carbon accumulation (ton C/ha/y)	Mean LEMMA carbon accumulation (ton C/ha/y)
ACR173	2246	123.4	115.3	82.1	2.3	1.3	0.7
ACR182	968	139.8	142.7	111.9	0.6	0.8	1.4
ACR189	641	157.1	128.6	96.1	3.3	0.6	0.9
ACR200	714	135.1	163.0	104.3	3.6	0.9	2.0
ACR262	5328	133.1	120.2	103.9	3.5	1.2	0.6
ACR282	6064	113.8	142.8	123.6	0.6	1.4	-0.6
ACR292	2202	127.8	175.4	110.6	1.3	-0.8	-1.2
ACR377	890	183.6	135.6	104.7	-2.0	0.4	-0.1
ACR378	782	207.7	122.6	97.8	-2.8	-0.1	0.2
CAR1013	7913	106.3	143.4	105.3	1.9	1.6	1.6
CAR1041	6863	110.1	121.5	114.1	2.5	0.3	-0.3
CAR1046	4593	98.0	106.0	106.6	-11.4	-1.1	3.4
CAR1066	5053	102.9	87.6	75.3	3.2	0.9	0.7
CAR1067	855	144.6	132.9	106.3	3.6	1.4	1.7
CAR1070	8411	133.7	130.4	106.8	0.3	0.5	-0.4
CAR1092	5917	47.1	51.8	41.6	0.6	0.2	0.6
CAR1095	6642	109.7	98.1	64.5	4.5	1.6	1.0
CAR1098	9624	122.2	164.6	108.4	4.1	1.1	1.7
CAR1099	5480	113.3	167.2	113.9	4.2	1.6	1.4
CAR1100	6439	124.7	221.7	138.6	0.1	3.4	4.1
CAR1102	1422	128.2	139.5	95.2	1.1	0.5	0.3
CAR1103	848	140.9	119.9	118.9	0.9	1.2	-0.3
CAR1104	1416	126.1	139.2	120.0	1.8	1.1	0.5
CAR1114	7837	108.3	94.7	105.9	-0.2	-1.1	-1.1
CAR1139	19418	146.3	157.4	109.4	3.9	1.0	0.7
CAR1140	7156	142.3	146.7	107.6	6.6	1.2	2.0
CAR1141	877	208.5	226.8	159.9	2.3	1.0	1.2

CAR1174	1637	123.8	135.7	93.1	1.6	0.9	-0.1
CAR1180	5005	152.4	140.4	115.1	2.7	0.3	-0.2
CAR1190	3491	141.5	168.2	113.5	0.0	0.3	2.0
CAR1191	8215	147.7	136.8	115.1	0.4	0.2	0.0
CAR1313	749	269.6	191.8	170.6	n/a	n/a	n/a
CAR1329	2547	n/a	n/a	n/a	n/a	n/a	n/a
CAR1330	935	n/a	n/a	n/a	n/a	n/a	n/a
CAR1339	13443	n/a	n/a	n/a	n/a	n/a	n/a
CAR1368	3240	n/a	n/a	n/a	n/a	n/a	n/a
CAR993	3100	136.8	136.7	103.9	4.4	0.7	0.5
**Area-weighted average**		**125.7**	**137.4**	**105.2**	**1.97**	**0.83**	**0.82**
**Standard error**		**4.61**	**5.94**	**3.49**	**0.54**	**0.16**	**0.22**

Table S3. Projects' landowner information

Project ID	Area (ha)	Offset Project Operator (OPO)	Category
ACR173	2246	Round Valley Indian Tribes	other
ACR182	968	Forest Carbon Partners, LP	other
ACR189	641	Hanes Ranch Inc.	other
ACR200	714	Edward Miller Trust	other
ACR262	5328	Edward Miller Trust	other
ACR282	6064	Western Rivers Forestry	other
ACR292	2202	Congaree River, LLC	other
ACR377	890	California Timberlands 2, LLC	other
ACR378	782	California Timberlands 2, LLC	other
CAR1013	7913	Sustainable Conservation, Inc.	other
CAR1041	6863	Sierra Pacific Industries	timber
CAR1046	4593	Trinity Timberlands, LLC	other
CAR1066	5053	Sierra Pacific Industries	timber
CAR1067	855	Berry Summit, LLC	other
CAR1070	8411	Yurok Tribe	other
CAR1092	5917	Sierra Pacific Industries	timber
CAR1095	6642	Coastal Forestlands, Ltd.	other
CAR1098	9624	The Conservation Fund	other
CAR1099	5480	The Conservation Fund	other
CAR1100	6439	The Conservation Fund	other
CAR1102	1422	Montesol, LLC	other
CAR1103	848	Ronald Glass	other
CAR1104	1416	GM Gabrych Family LP	other
CAR1114	7837	Sierra Pacific Industries	timber
CAR1139	19418	Usal Redwood Forest Company, LLC	other
CAR1140	7156	Coastal Ridges LLC	other
CAR1141	877	Fred M. van Eck Forest Foundation	other
CAR1174	1637	Eddie Ranch Properties, LLC	other
CAR1180	5005	Mailliard Ranch	other
CAR1190	3491	Mendocino Redwood Company, LLC	timber
CAR1191	8215	Mendocino Redwood Company, LLC	timber
CAR1313	749	Save the Redwoods League	other
CAR1329	2547	Hunter Ranch LLC	other

CAR1330	935	Bohemian Club	other
CAR1339	13443	Green Diamond Resource Company	timber
CAR1368	3240	California Timberlands 2, LLC	other
CAR993	3100	Yurok Tribe	other

Table S4. Additionality criteria per project. We rate each project as passing (✓) or failing (✗) our criteria as established in Table 1. While the specific details of any individual project are fairly uncertain and should not be scrutinized, this demonstrates a general pattern that the portfolio of projects does not show strong evidence of sequestering additional carbon. "-" indicates that the project was not evaluated on the given criteria, due to having less than three years since initiation or being outside the domain considered.

Project ID	**1. Pre-project carbon**<br>Historical carbon accumulation rate has been near-zero or negative; flat baseline is a realistic BAU	**2. Pre-project harvest**<br>Project areas were harvested at similar rates as other similar forests over the historical period	**3. Pre-project species**<br>Project areas have similar tree species to nearby forests, or have more high-value species (for Northern Coast)	**4. Post-project carbon**<br>Carbon accumulation rate after project initiation is greater than the pre-initiation and that of similar forests	**5. Post-project carbon**<br>Harvesting rate has decreased relative to pre-project levels and relative to similar forests
ACR173	✗	✓	-	✓	✓
ACR182	✗	✓	✗	✗	✓
ACR189	✗	✓	-	✗	✓
ACR200	✗	✓	✓	✗	✗
ACR262	✗	✓	✗	✗	✗
ACR282	✗	✓	✗	-	-
ACR292	✗	✗	✗	-	-
ACR377	✗	✓	-	-	-
ACR378	✗	✓	-	-	-
CAR1013	✗	✓	✗	✗	✓
CAR1041	✗	✓	-	✓	✓
CAR1046	✗	✗	-	✗	-
CAR1066	✓	✓	-	✓	✓
CAR1067	✗	✓	-	✗	✓

CAR1070	✗	✓	-	-	-
CAR1092	✓	✗	-	✗	✗
CAR1095	✗	✗	-	✗	✗
CAR1098	✗	✓	✗	-	-
CAR1099	✗	✓	✗	-	-
CAR1100	✗	✗	✓	-	-
CAR1102	✗	✓	✗	-	-
CAR1103	✗	✓	-	✓	✓
CAR1104	✗	✓	-	✗	✗
CAR1114	✓	✓	-	✗	✗
CAR1139	✗	✓	✗	-	-
CAR1140	✗	✓	✗	-	-
CAR1141	✗	✓	✓	✗	✗
CAR1174	✗	✓	-	-	-
CAR1180	✗	✗	✓	-	-
CAR1190	✗	✓	✓	-	-
CAR1191	✗	✓	✗	-	-
CAR1313	✗	✓	✓	-	-
CAR1329	✗	✓	-	-	-
CAR1330	✗	✗	✗	-	-
CAR1339	✗	✓	✗	-	-
CAR1368	✗	✓	-	-	-
CAR993	✗	✓	-	✗	✓
